# Bacterial endophytes suppress collar rot of chickpea caused by *Sclerotium rolfsii* through antagonism and defense enzyme induction

**DOI:** 10.3389/fmicb.2026.1867487

**Published:** 2026-09-24

**Authors:** Manisha Kumari, Meenakshi Arya, Jonnada Likhita, Yashowardhan Singh, Priyanshi Garg, Amita Malik, Anshuman Singh, Maharishi Tomar, Rashid Mumtaz Khan, Prashant Jambhulkar, Sayed Sartaj Sohrab, S. K. Chaturvedi, B. K. Behera

**Affiliations:** 1 Rani Lakshmi Bai Central Agricultural University, Jhansi, India; 2 ICAR-National Research Institute for Integrated Pest Management, New Delhi, India; 3 Janta Vedic College, Baraut, Uttar Pradesh, India; 4 ICAR-Indian Agricultural Statistic Research Institute, New Delhi, India; 5 ICAR-Central Institute of Post-Harvest Engineering & Technology, Ludhiana, India; 6 Department of Chemistry, College of Science, Qassim University, Buraidah, Saudi Arabia; 7 Special Infectious Agents Unit, King Fahd Medical Research Center, King Abdulaziz University, Jeddah, Saudi Arabia; 8 Department of Medical Laboratory Sciences, Faculty of Applied Medical Sciences, King Abdulaziz University, Jeddah, Saudi Arabia; 9 National Fisheries Development Board, Hyderabad, India

**Keywords:** 16S rRNA sequencing, chickpea, defense enzymes, endophytic bacteria, *Sclerotium rolfsii*

## Abstract

**Introduction:**

Collar rot of chickpea, caused by the soil-borne pathogen *Sclerotium rolfsii*, is commonly managed with chemical fungicides, which may pose environmental and health risks. Bacterial endophytes offer a sustainable alternative through pathogen antagonism and induction of host defense responses.

**Methods:**

Seventy-three bacterial endophytes isolated from diverse plant sources were screened against *S. rolfsii* under laboratory conditions. Promising isolates were further evaluated in pot culture through seed priming and soil drenching. Their biocontrol efficacy, plant growth promotion, defense enzyme activities, and biochemical and molecular characteristics were assessed.

**Results:**

Three isolates, RLB E-30, RLB E-25 and RLB E-64, showed strong antagonistic activity. RLB E-30 recorded the highest mycelial inhibition (82.60%). Seed priming and soil drenching with RLB E-30 resulted in 92.58% seed germination and reduced total disease mortality to 19.43% compared with the untreated control. The isolates were identified as *Alcaligenes faecalis* RLB E-30, *Bacillus velezensis* RLB E-25 and *Acinetobacter schindleri* RLB E-64. Endophyte application enhanced defense-related and antioxidant enzyme activities and promoted plant growth and root development.

**Conclusion:**

*Alcaligenes faecalis* RLB E-30 showed the greatest potential for managing chickpea collar rot through direct antagonism and induction of host defense responses. Its combined biocontrol and plant growth-promoting effects highlight its potential as an environmentally sustainable alternative to chemical disease management.

## Introduction

Chickpea (*Cicer arietinum* L.) is one of the most important food legumes cultivated worldwide and is a major source of protein, minerals and dietary fiber, particularly in South Asia. It ranks as the third most extensively cultivated legume globally after common bean and field pea (Mageshwaran et al., 2022). Chickpea is grown over an area of about 14.56 million hectares with an average productivity of 970 kg ha^−1^ (Merga and Haji, 2019). As per the 2nd Advance Estimates (DA & FW, 2025–26), India's pulse output is projected at 238.69 lakh tons and chickpea is expected to make up about 50%, nearly to 117.92 lakh tons in production. India is the largest producer of chickpea, contributing approximately 64% of global production, with nearly 30% of the national pulse acreage under chickpea cultivation, accounting for about 38% of total pulse production. Major chickpea-producing states include Madhya Pradesh, Maharashtra and Rajasthan, which together contribute more than 95% of the country's production (Chiranjeevi et al., 2020; Yaremko et al., 2024). Despite its agronomic and nutritional importance, chickpea productivity is severely constrained by several biotic stresses, particularly soil-borne diseases.

Among soil-borne diseases, collar rot caused by *Sclerotium rolfsii* Sacc. is one of the most destructive diseases of chickpea, especially during the seedling and early vegetative stages. The pathogen is seed and soil-borne and causes severe plant mortality under favorable environmental conditions (Manjunatha et al., 2022). Collar rot can result in plant mortality of up to 54.79% in India (Shirsole et al., 2019; Kumar et al., 2025). Initial symptoms include girdling and rotting at the collar region, followed by yellowing, wilting and eventual death of the plant (Bagul et al., 2024; Kumar et al., 2025). Disease development is highly influenced by environmental factors, particularly warm soil temperatures ranging from 25 to 30 °C and high soil moisture levels of around 80%, which favor pathogen growth and symptom expression (Tamang and Saha, 2023). As the disease progresses, infected plants often exhibit rapid wilting and tan-colored lesions at the stem–soil interface, resulting in significant yield losses (Okon et al., 2025). It has been reported that for every 1% increase in disease severity, chickpea yield may decline by approximately 0.13 q ha^−1^ (Tamang and Saha, 2023).

Management of collar rot disease has largely depended on the use of chemical fungicides. Although fungicides are effective in suppressing disease incidence, their indiscriminate and prolonged use poses serious environmental and ecological concerns, including soil and water contamination and adverse effects on non-target organisms (Kumari et al., 2020). Moreover, excessive reliance on chemical fungicides can lead to the development of resistant pathogen strains, thereby reducing their long-term efficacy (Islam et al., 2024). The use of resistant cultivars is considered an environmentally sustainable strategy; however, resistance is often short-lived due to the complex genetic interactions between host plants and rapidly evolving pathogen populations (Pati et al., 2025; Aslam et al., 2026). These limitations emphasize the need for alternative, eco-friendly disease management strategies.

Biological control offers a sustainable and environmentally benign alternative to chemical fungicides by exploiting natural microbial interactions such as competition, antibiosis, enzyme production and induction of host defense responses (Selari et al., 2022). Among the various biological control agents, bacterial endophytes have gained increasing attention due to their ability to colonize internal plant tissues without causing any apparent harm and their potential to enhance plant growth and disease resistance (Muthu Narayanan et al., 2022). Bacterial endophytes enter plant tissues through roots, stomata, or natural openings and establish stable populations within the host by producing cell wall–degrading enzymes such as cellulases and pectinases (Bharadwaj, 2024). Unlike rhizospheric bacteria, endophytic bacteria reside within plant tissues, providing them with a protected ecological niche and enabling sustained interaction with host metabolic and defense pathways (Santoyo et al., 2016).

Endophytic bacterial colonization strategies share similarities with those of pathogenic microbes, including motility, immune response modulation and metabolic adaptation to the host environment (Wippel, 2023). The rhizosphere and phyllosphere serve as major sources of endophytic microbial communities, with a high degree of similarity reported between microbial populations in these regions and those residing within plant tissues (Rajendran et al., 2023). Endophytic bacteria are known to produce a wide range of antimicrobial metabolites and volatile organic compounds that inhibit soil-borne pathogens such as *Fusarium* spp., *Rhizoctonia* spp., and *Sclerotium* spp., thereby reducing disease incidence and severity (Eke et al., 2023; Rabbee et al., 2024; Serrão et al., 2024). Additionally, these bacteria can induce systemic resistance in host plants by activating pathogenesis-related proteins and defense-related enzymes, thereby enhancing the plant's innate defense mechanisms (Mageshwaran et al., 2022).

Microbial inoculants, including endophytic bacteria, play a critical role in host–pathogen interactions by triggering plant defense signaling pathways and enhancing resistance against pathogen invasion (Sahu et al., 2022; Mageshwaran et al., 2022). Previous studies have demonstrated that bacterial genera such as *Bacillus, Serratia*, and *Pseudomonas* significantly reduce disease severity in chickpea through defense induction and pathogen suppression (Abdullaeva et al., 2024; Hardiyanti et al., 2024). Several reports have documented the isolation of bacterial endophytes and their antagonistic activity against fungal pathogens (Manjunatha et al., 2022; Ozturk and Endes, 2023; Bhargavi et al., 2024). However, information on the use of bacterial endophytes for the management of collar rot disease of chickpea caused by *S. rolfsii* remains limited, particularly with respect to their role in inducing host defense responses. Considering the economic importance of chickpea, the destructive nature of collar rot disease and the biocontrol potential of bacterial endophytes, it is imperative to explore their role in the management of *S. rolfsii*. The present study was therefore undertaken to isolate and characterize bacterial endophytes from chickpea, evaluate their antagonistic activity against *S. rolfsii* (SR) and assess their efficacy in suppressing collar rot disease under pot culture conditions. In addition, the study aimed to elucidate the role of selected endophytic bacteria in inducing defense-related enzymes in chickpea, thereby contributing to sustainable disease management strategies. Biological management practices adopted so far are not very efficient to manage the wide spread of collar rot disease in chickpea as the host range of the pathogen is very broad and no resistant sources are available for this disease in chickpea. Limited information is available on characterization of endophytic bacterial strains from different crops including chickpea and growth environments to find effective candidates for collar rot management (Bhargavi et al., 2024).

## Material and methods

### Collection and sub-culturing of the pathogen

The pathogen culture was collected from the Department of Plant Pathology, Rani Lakshmi Bai Central Agricultural University (RLBCAU), Jhansi and sub-cultured on Potato Dextrose Agar (PDA) plates and incubated at 28 ± 2 °C for 5–7 days. The culture of the pathogen was purified by a single sclerotial culture on PDA plates which was then observed under the microscope and identified by comparing with the hyphal characters of *S. rolfsii*. The identified culture was maintained at 4 °C for further studies. Further, the pathogenicity of the pathogen was conducted to prove the Koch postulates.

### Isolation of endophytic bacteria

Seventy-three samples were collected from the farms and fields of RLBCAU (25.51 °N latitude and 78.56 °E longitude at an altitude of 227 m above Mean Sea Level) in Jhansi, Uttar Pradesh, India, to isolate bacterial endophytes. The collected samples were washed with sterilized distilled water and tissue samples were cut into small bits and disinfected with 1% sodium hypochlorite solution. The endophytic bacteria (EB) were isolated from different plant parts using the protocol described by Sahu et al. (2020) with minor modifications. The sterilized bits were crushed in 2 mL of sterile distilled water and 1 mL of crude extracts was serially diluted up to 10^−4^ dilutions. The dilutions were spread on Nutrient Agar (NA) plates and incubated at 28 ± 2 °C for 24–48 h. After incubation, morphologically different bacterial colonies were selected and streaked on fresh NA plates. All selected isolates were sub-cultured on NA slants and preserved in a refrigerator at 4 °C for further study. The isolated EBs were assigned respective codes for further research.

### Validation of surface sterilization

For ensuring that only endophytic bacteria were isolated, the effectiveness of the surface sterilization procedure was validated. After the final rinse of the plant samples, an aliquot of the sterile distilled water used in the last washing step was spread onto Nutrient Agar (NA) plates and incubated at 28 ± 2 °C for 48–72 h. Additionally, the surface-sterilized plant bits were gently imprinted onto NA plates before tissue maceration. The absence of microbial growth on both the rinse-water plates and imprint plates confirmed the successful removal of epiphytic microorganisms from the plant surface. Therefore, the bacterial isolates recovered from the plant tissues were considered true endophytes (Sahu et al., 2022; Srivastava et al., 2024).

### Antagonistic activity of endophytic bacteria against *S. rolfsii* by dual and inverted plate assay

All isolated EBs were tested for antifungal activity using the dual culture assay and inverted plate technique (Singh and Gaur, 2016; Bhargavi et al., 2024). The mycelial disc of the fresh culture was placed on the PDA plate and the EB was streaked 1 cm away from it and the plates were incubated at 28 °C for 72 h. A control PDA plate containing a mycelial disc of SR was maintained as a control. The experiment was performed in three replications for each bacterium and control. The inhibition of mycelial growth was observed after 5–7 days and the diameter of the zone of inhibition was measured

The isolates that showed inhibition against SR in dual culture assay were further tested by the inverted plate method. The quantitative assessment of inhibitory volatile compounds produced *in vitro* by selected effective endophytes against *S. rolfsii* was conducted following the method described by Gupta et al. (2022). Briefly, 0.1 mL of the selected bacterial broth culture was plated on nutrient agar medium, while the mycelium of *S. rolfsii* (5 mm plug) from the actively growing culture was cut and placed at the center of the fresh PDA plate. The partitioned plates containing only fungal mycelium, but no bacteria, served as a control. The PDA plate containing the mycelial plug was kept inverted over the nutrient agar plate with bacterial culture and sealed firmly with parafilm. The plates were incubated at 28 ± 2 °C for 7 days and the inhibition of fungal growth was measured. The inhibition of pathogen growth was calculated as the percentage of extension of hyphae on the control plates in the absence of endophytes (Sornakili et al., 2020).

### *In-vivo* plant growth promotion assessment

Pot culture experiments under controlled conditions have been used to evaluate the antagonistic potential of different EB treatments in plant growth promotion and disease suppression of *S. rolfsii*. To have uniform pathogen pressure, the inoculum of *S. rolfsii* was mass-multiplied on the sorghum grain medium. Sorghum grains (5 kg) were thoroughly washed with tap water, soaked for 5–6 h, drained and air-dried for 2–3 h to get rid of excess moisture (Figure 1). The grains were then put into 500 mL conical flasks and sterilized twice at 15 lbs pressure (121.5 °C) for 20 min. After cooling, the sterilized grains were inoculated with 4 to 5 mycelial disks from the actively growing *S. rolfsii* cultures under aseptic conditions and incubated at 28 ± 2 °C for 7–10 days. The flasks were shaken daily to facilitate uniform colonization and dense mycelial growth, acted as inoculum (Hossain et al., 2013; Shirsole et al., 2019; Shriganesh, 2023).

**Figure 1:**
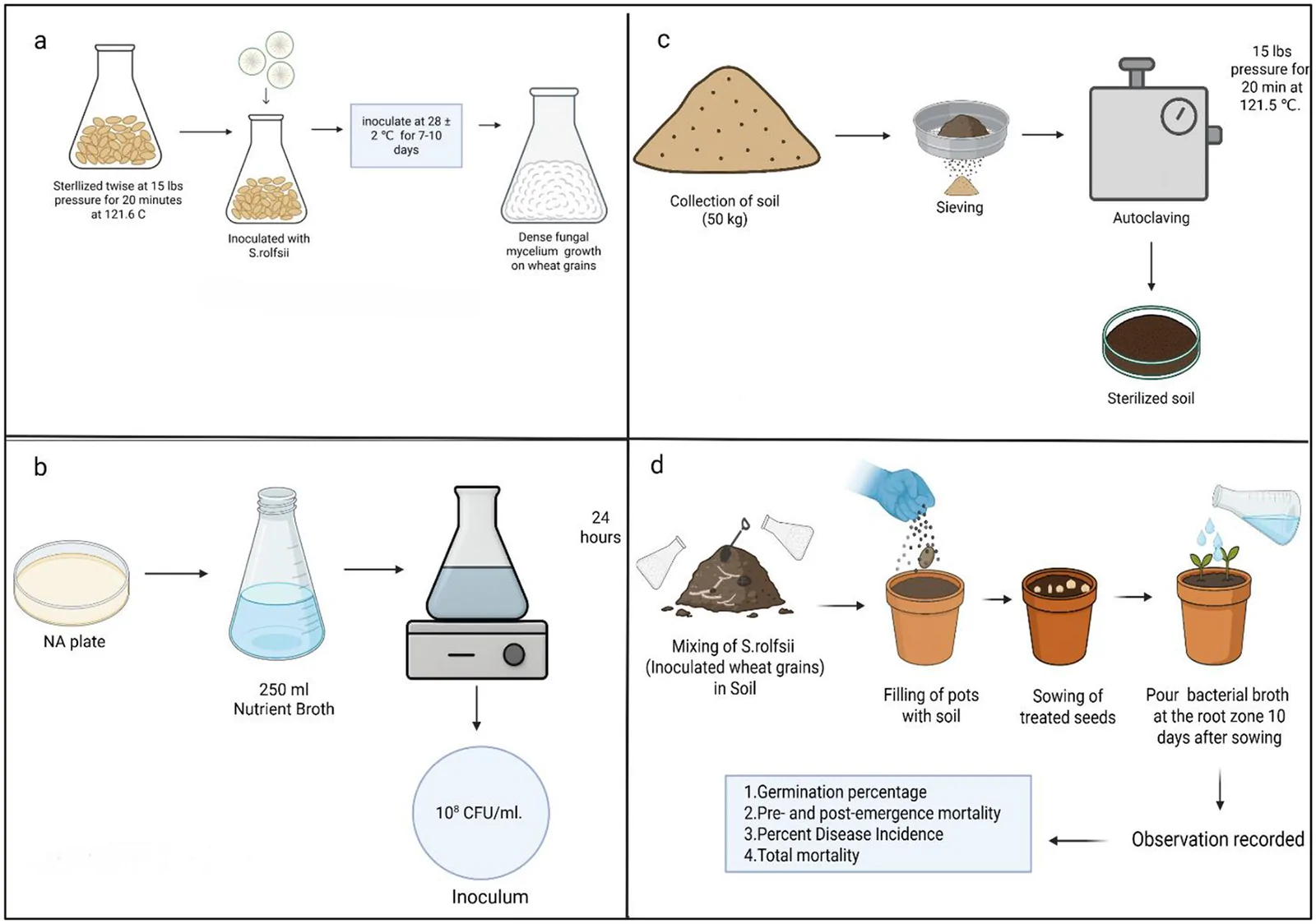
**(a)** Mass multiplication of wheat grains **(b)** Inoculum of endophytic bacteria **(c)** Autoclaving of the soil **(d)** Preparation of Sick Soil and Sowing of seeds.

The inoculum of endophytic bacteria was prepared by inoculating the culture on NA plates into conical flasks containing 250 mL nutrient broth, which were inoculated for 24 h placed in an orbital shaker at 200 rpm and incubated for 2 days. The concentration of 1 × 108 CFU was estimated using the standard serial dilution and plate count method. Briefly, the culture suspension was serially diluted and appropriate dilutions were plated on nutrient agar. After incubation, the number of colonies formed was counted and the CFU was calculated by multiplying the colony count with the corresponding dilution factor, which resulted in an approximate concentration of 1 × 108 CFU mL^−1^ (Figure 1).

The 50 Kg soil was collected from the agricultural farm, RLBCAU, Jhansi and was sieved through a large sieve to get the fine soil. The soil was autoclaved twice at 15 lbs pressure for 20 min at 121.5 °C (Figure 1). The small plastic pots measuring 22.5 cm x 19 cm were filled with 1 kg of the sterilized soil mixed with 5 g of *S. rolfsii* culture grown on wheat grains in all pots except control. Nine plants per replication were used and the variety selected for this study was Annigeri (Figure 1). The inoculum of the antagonistic EB was multiplied for all experiments by harvesting the cells from nutrient broth cultures grown at 28 ± 2 °C for 48 h followed by centrifugation at 6,000 rpm for 25 min. The inoculum was re-suspended in sterile distilled water, after which the concentration was adjusted using a spectrophotometer to 10^8^ CFU/mL. These suspensions were further used for the seed treatment method (Shukla et al., 2022; Akram et al., 2024).

Seed priming was done using the respective EB by adopting the method given by Akram et al. (2024). The seeds were surface sterilized using 1% NaOCl followed by washing with ethanol and finally twice with sterile distilled water (SDW) to remove traces of NaOCl. The seeds were dried in a laminar flow for 4–5 h and were primed with EB. Seeds were soaked in bacterial broth (for seed priming) or distilled water (for the control) for 12 h and 9 seeds were sown into the pot containing sterilized soil. For the treatment where only drenching was taken up, 12 seeds (not primed) per pot were sown and after germination of the seedlings (10 days), 100 mL bacterial broth was applied at the root zone. A moisture level of up to 80% was also ensured, as this pathogen favors high moisture. Observations on percent disease incidence were taken post-pathogen inoculation as per the technical details. Each treatment was replicated thrice.

Observations were recorded at 10, 20, 25, 35, and 45 days after sowing (DAS). The trait parameters evaluation included germination percentage (Senthilkumar et al., 2009), pre- and post-emergence mortality (Patel et al., 2020) and per cent Disease Incidence (Senthilkumar et al., 2009). Observation recorded as per the given formula. In this study, 11 treatments were taken up with the following treatment details: T1—seed priming with EB 1; T2- seed priming with drenching with EB 1; T3—soil drenching with EB 1; T4—seed priming with EB 2; T5—seed priming and drenching with EB 2; T6—soil drenching with EB 2; T7—seed priming with EB 3; T8—seed priming and drenching with EB 3; T9—soil drenching with EB 3; T10—seed treatment with Mancozeb (3 g/kg of seed) and T11 was control. Each treatment was replicated thrice and the technical details of the experiment is represented in Table 1.


$$\text{Germination }(\%)\text{=}\frac{\text{Total no}.\text{ of seeds germinated}}{\text{Total no}.\text{ of seeds sown}}\times \;\text{100}$$

$$\begin{array}{l} \text{Percent disease incidence }(\text{PDI})\\ =\frac{\text{Total no}.\text{ of infected plants}}{\text{Total no}.\text{ of plants}}\times \;\text{100} \end{array}$$

$$\begin{array}{l} \text{Pre-emergence mortality }(\%)\\ =\frac{\text{No}.\text{ of ungerminated seeds}}{\text{No}.\text{ of seeds sown}}\times \;\text{100} \end{array}$$

$$\begin{array}{l} \text{Post-emergence mortality }(\%)\\ =\frac{\text{No}.\text{ of seedlings collapsed}}{\text{Total no}.\text{ of seedling emerged}}\times \;\text{100} \end{array}$$

$$\begin{array}{l} \text{ Total mortality }(\%)\\ =\text{pre-emergence mortality}(\%)\\ +\text{post-emergence mortality}\;(\%) \end{array}$$

**Table 1:** Technical details of the experiment.

Sl. no.	Details of the experiment	
1	Year and season of experiment	2023–24, Rabi
2	Statistical design	Completely Randomized Design (CRD)
3	No. of replications	03
4	No. of treatments	11
5	No. of plants per replication	09
6	Variety used	Annigeri
7	Observation to be recorded	10, 20, 25, 35, and 45 days after sowing (DAS)
8	Trait parameters to be evaluated	Germination percentage, Pre and Post emergence mortality, Percent Disease Incidence (PDI)

### Biochemical and molecular characterization of effective endophytic bacteria

The biochemical characterization of the bacterial endophytes was assessed by Biochemical Test Kit (KB001 HiMViC™). The reaction of the effective EB was recorded based on the color change. All the tests except indole, Methyl red and Voges–Proskauer were performed by following McDevitt (2009) methods.

For molecular characterization, bacterial genomic DNA was extracted using the CTAB protocol described by Williams et al. (2004). For DNA extraction, the isolated EBs were grown in nutrient broth at 28 ± 2 °C for 24 h with shaking at 220 rpm. The cultures were then centrifuged at 10,000 rpm for 5 min at room temperature and the supernatant was discarded. The pellet was washed twice with Tris-EDTA buffer, then lysozyme and RNase were added and the mixture was incubated at 37 °C for 30 min. SDS and proteinase K were added and incubated at 55 °C for 90 min. Sodium Chloride and phenol: chloroform: isoamyl alcohol were added and incubated at room temperature for 30 min. The upper aqueous phase was transferred to a fresh tube and sodium acetate and chilled isopropanol were added and incubated at −20 °C for 1 h. The tubes were centrifuged at 10,000 rpm for 10 min at 4 °C. The supernatant was discarded and the pellet was washed twice with chilled ethanol (70%). The pellet was air-dried until the ethanol smell disappeared and suspended in 40–50 μL TE buffer and stored at 4 °C for further use. The isolated EBs were identified based on the amplification of the 16s rRNA gene. The PCR amplification of the 16s rRNA gene was done using the Go-Taq Green master mix (M/s Promega, United States). The primer pair used in the PCR was: forward primer 27F (AGAGTTTGATCCTGGCTCAG) and reverse primer 1,492R (TACGGTTACCTTGTTACGACT). The PCR amplification for 16s rRNA (35 cycles) was done using a thermocycler (Eppendorf) and the PCR conditions were as follows: heat lid temperature 110 °C, initial denaturation temperature 94 °C for 4 min, denaturation temperature 94 °C for 30 s, annealing temperature 52 °C for 45 s, extension temperature 72 °C for 90 s and final extension temperature 72 °C for 10 min. The PCR product was run in 1.2% agarose gel electrophoresis along with a 1 kb DNA ladder (Hi Media; Figure 2).

**Figure 2:**
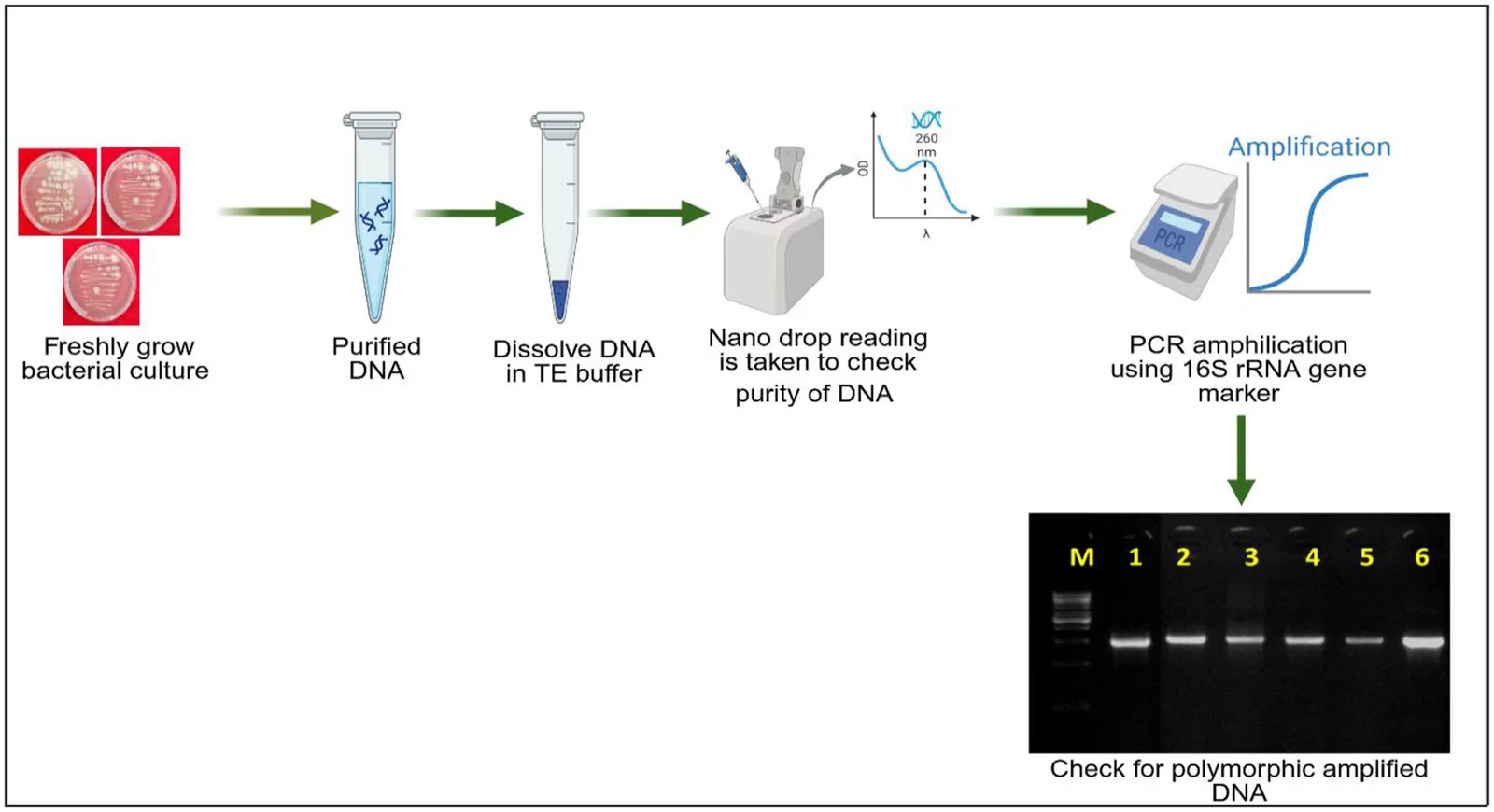
Workflow for molecular identification of endophytic bacterial isolates using 16S rRNA gene amplification.

### DNA sequencing and phylogenetic analysis of the effective endophytic bacteria

The PCR products were amplified and sent for sequencing to Medauxin, Bengaluru, India. The obtained sequences were subjected to the BLASTn search program of the NCBI, for a similarity search with already available 16S rRNA nucleotide sequences of related species available in GenBank NCBI. All the sequences were aligned using the CLUSTAL W multiple sequence alignment program. Phylogenetic trees were constructed by using Mega 11 software using the Neighbor-joining method with a 1,000 bootstrap value (Tamura et al., 2021; Bhargavi et al., 2024).

### Effect of endophytic bacteria inoculation on defense-related enzymes

The estimation of defense-related enzymes *viz.*, SOD (superoxide dismutase), Peroxidase (POX), APX (ascorbate peroxidase), CAT (catalase) and PPO (polyphenol oxidase) were analyzed at pre and post inoculation stages to evaluate the effect of endophytes in inducing systemic resistance (ISR). The protocol as described was repeated to keep the pots for all the treatments except that the number of seeds sown for this experiment was 9 per pot. Three to four plants were collected from the respective treatments each at pre (at 10 days) and post inoculation stages (20 days) and stored at −20 °C to stop the enzymatic activities before further analysis. The treatment details of the experiment are mentioned in the Table 2.

**Table 2:** Treatment details of the experiment for defense-related enzymes.

Sl. no.	Treatment no.	Details
1	T1	Seed priming with EB 1
2	T2	Seed priming + Drenching with EB 1
3	T3	Soil drenching with EB 1
4	T4	Seed priming with EB 2
5	T5	Seed priming + Drenching EB 2
6	T6	Soil drenching with EB 2
7	T7	Seed priming with EB 3
8	T8	Seed priming +Drenching EB 3
9	T9	Soil drenching with EB 3
10	T10	Seed treatment with Mancozeb @ 3 g/kg seed
11	T11	Control

Super Oxide Dismutase (SOD) activity was evaluated in both the test samples (pre- and post-inoculation) and the control using the method described by Beauchamp and Fridovich (1971). A 100 mg test sample was homogenized in 50 mM phosphate buffer and centrifuged for 15 min. The supernatant was used as the enzyme extract. Fresh glass test tubes were prepared and labeled and reagents were added to each tube. The final volume was increased to 2.8 mL by adding 50 mM phosphate buffer and 75 μM riboflavin. The tubes were vortexed and exposed to a light source. The tubes were illuminated for 30 min and then covered with a black cloth. Two controls were prepared: one blank sample (B1), which was exposed to light and had maximal color and another blank sample (B2), which was kept in the dark and had no enzyme extract. For the estimation of Peroxidase activity, follow the method of Castillo et al. (1984) with slight modifications. A 100 mg sample was homogenized in 10 mL of 50 mM phosphate buffer (pH 7.0) and centrifuged at 10,000 rpm for 15 min. The supernatant was collected and used as the enzyme extract. In a separate test tube, the following reagents were added sequentially: 1 mL of 100 mM phosphate buffer (pH 6.1), 0.5 mL of guaiacol, 0.5 mL of hydrogen peroxide (H_2_O_2_) and 0.9 mL of water. Following this, 100 μL of the enzyme extract was introduced into the mixture. The absorbance of the reaction mixture was measured at 470 nm at 15-s intervals over 3 min using a spectrophotometer. The peroxidase activity was quantified by calculating the change in absorbance per minute (ΔOD/min) per milligram of protein. This measure indicates the enzyme's activity in the test samples.

Ascorbate peroxidase (APX) activity was immediately assessed in fresh extracts from all resistant and susceptible genotypes, including inoculated and uninoculated controls. The enzyme activity was determined using the method outlined by Nakano and Asada (1981). The sample was homogenized in 50 mM phosphate buffer and centrifuged for 15 min to obtain the enzyme extract and transferred to a test tube, along with 100 μL of phosphate buffer, 0.5 mL of ascorbic acid, 0.5 mL of EDTA solution and 0.1 mL of hydrogen peroxide. The reaction was monitored by measuring the decrease in absorbance at 290 nm every 30 s for 3 min. The amount of ascorbate oxidized was calculated using the molar extinction coefficient and enzyme activity was expressed as nanomoles of ascorbate oxidized per milligram of protein per minute. Catalase activity was determined following the method described by Aebi (1984). The 150 mg sample was homogenized in 50 mM phosphate buffer (6 mM) and centrifuged for 15 min to obtain the enzyme extract and transferred to a test tube and mixed with 100 mM phosphate buffer. The reaction was initiated by adding 75 mM H_2_O_2_. The enzyme activity was calculated using the molar extinction coefficient for hydrogen peroxide and expressed in micromoles of H_2_O_2_ decomposed per milligram of protein per minute. This metric provides a quantitative measure of the enzyme's ability to catalyze the breakdown of hydrogen peroxide in the test samples. The enzyme activity was quantified by measuring in absorbance at 240 nm using a spectrophotometer. Polyphenol oxidase (PPO) enzymatic activity was evaluated method described by Kumar et al. (2018). The PPO activity was quantified by monitoring the increase in absorbance at 420 nm, which corresponds to the oxidation of catechol by PPO, reflecting the enzyme's activity in the samples. A 150 mg portion of the test sample was homogenized in 6 mL of phosphate buffer using a mortar and pestle. The homogenate was then centrifuged at 10,000 rpm for 20 min. The resulting supernatant was collected and used as the enzyme extract. For the PPO activity assay, 500 μL of the enzyme extract was added to a test tube, followed by 700 μL of sodium phosphate buffer (pH 6.0) and 100 μL of 0.2 M catechol. The absorbance of the mixture was measured at 420 nm using a spectrophotometer. The rate of increase in absorbance was monitored over 3 min, with readings taken every 30 s. The rate of absorbance increase was compared against a standard curve generated using catechol. The enzyme activity was then expressed as units of PPO per milligram of protein, providing a measure of the enzyme's ability to oxidize catechol in the test samples.

### Statistical analysis

Statistical analysis was carried out wherever it was necessary by using WASP (Web Agri Stat Package) version 2.0 software.

## Results

### Collection and sub-culturing of the pathogen *Sclerotium rolfsii*

The collected culture of *S. rolfsii* was sub-cultured on PDA and incubated until full growth was observed. A whitish fan-shaped colony developed sclerotial bodies after 3–4 days, initially white but later brown. The fungus was identified by observing mycelial characters under a microscope at 40X magnification, which showed coarse mycelial strands giving a ropy appearance. From the pathogenicity study, it was confirmed that *S. rolfsii* caused collar rot disease and the pathogen was successfully re-isolated from the inoculated chickpea plants thereby fulfilling Koch's postulates.

### *In vitro* evaluation of selected endophytes for antagonistic activity by dual culture and inverted plate bioassay

A total of 73 EB was isolated and tested for antagonistic activity against *Sclerotium rolfsii* by the dual culture assay (Table 3). Out of 73 endophytic bacterial isolates, three isolates *viz.*, RLB E-25, RLB E-30 and RLB E-64 were found to be effective in managing the *S. rolfsii* under dual culture assay by inhibiting the growth of the fungus from 54.33 to 82.60% (Figure 3). Out of these, the maximum inhibition rate was shown by RLB E-30 of 73.33, 79.16, 79.71, and 82.60% at 24, 48, 72, and 96 h, respectively followed by RLB E-64 where the inhibition range varied from 64.46 to 80.74% from 24 to 96 h. Similarly, isolate RLB E-25 was found to inhibit the growth of *S. rolfsii* from 63.33 to 74.07% at 24 to 96 h (Table 4; Figure 3). The antifungal volatile activity of different EB against *S. rolfsii* was also assessed using an inverted plate bioassay. Among the three effective EB tested (RLB E-25, RLB E-30, and RLB E-64), RLB E-30 exhibited the highest inhibition rates. Specifically, it showed per cent inhibition rates of 80.00, 91.34, 89.67, and 90.74% at 24, 48, 72, and 96 h, respectively. RLB E-64 also demonstrated significant antifungal activity, with inhibition rates ranging from 71.10 to 81.22%. In comparison, RLB E-25 displayed a lower inhibition range, from 66.00 to 69.88% (Table 5).

**Table 3:** Total number of endophytic bacteria isolated from different plant parts.

Sl. no.	Source	Isolate code	No. of isolates
1	Rice root	RLB E-1, RLB E-2, RLB E-3, RLBE-4	4
2	Rice leaf	RLB E-5, RLB E-6	2
3	Rice stem	RLB E-7, RLB E-8	2
4	Chickpea root	RLB E-9, RLB E-10, RLB E-56, RLB E-57	4
5	Chickpea stem	RLB E-11, RLB E-12, RLB E-13, RLB E-14	4
6	Chickpea leaf	RLB E-15, RLB E-16, RLB E-17, RLB E-18, RLB E-19	5
7	Taro root	RLB E-20	1
8	Calotropis root	RLB E-21	1
9	Lawn grass root	RLB E-22, RLB E-23, RLB E-43, RLB E-44	4
10	Mungbean leaf	RLB E-24, RLB E-25, RLB E-26,	3
11	Mungbean root	RLB E-27	1
12	Spinach leaf	RLB E-28, RLB E-29, RLB E-30, RLB E-31	4
13	Bean leaf	RLB E-32, RLB E-33, RLB E-34	3
14	Bean root	RLB E-35, RLB E-36, RLB E-37, RLB E-38	4
15	Bean stem	RLB E-39, RLB E-40, RLB E-41, RLB E-42	4
16	Mustard root	RLB E-45, RLB E-46, RLB E-47, RLB E-48, RLB E-49	5
17	Mustard leaf	RLB E-50, RLB E-51, RLB E-52, RLB E-53	4
18	Mustard stem	RLB E-54, RLB E-55	2
19	Okra root	RLB E-58, RLB E-59	2
20	Tomato leaf	RLB E-60, RLB E-61	2
21	Onion leaf	RLB E-62, RLB E-63, RLB E-64, RLB E-65	4
22	Onion root	RLB E-66, RLB E-67	2
23	Wheat stem	RLB E-68, RLB E-69, RLB E-70	3
24	Wheat root	RLB E-71, RLB E-72, RLB E-73	3
Total isolates			**73**

**Figure 3:**
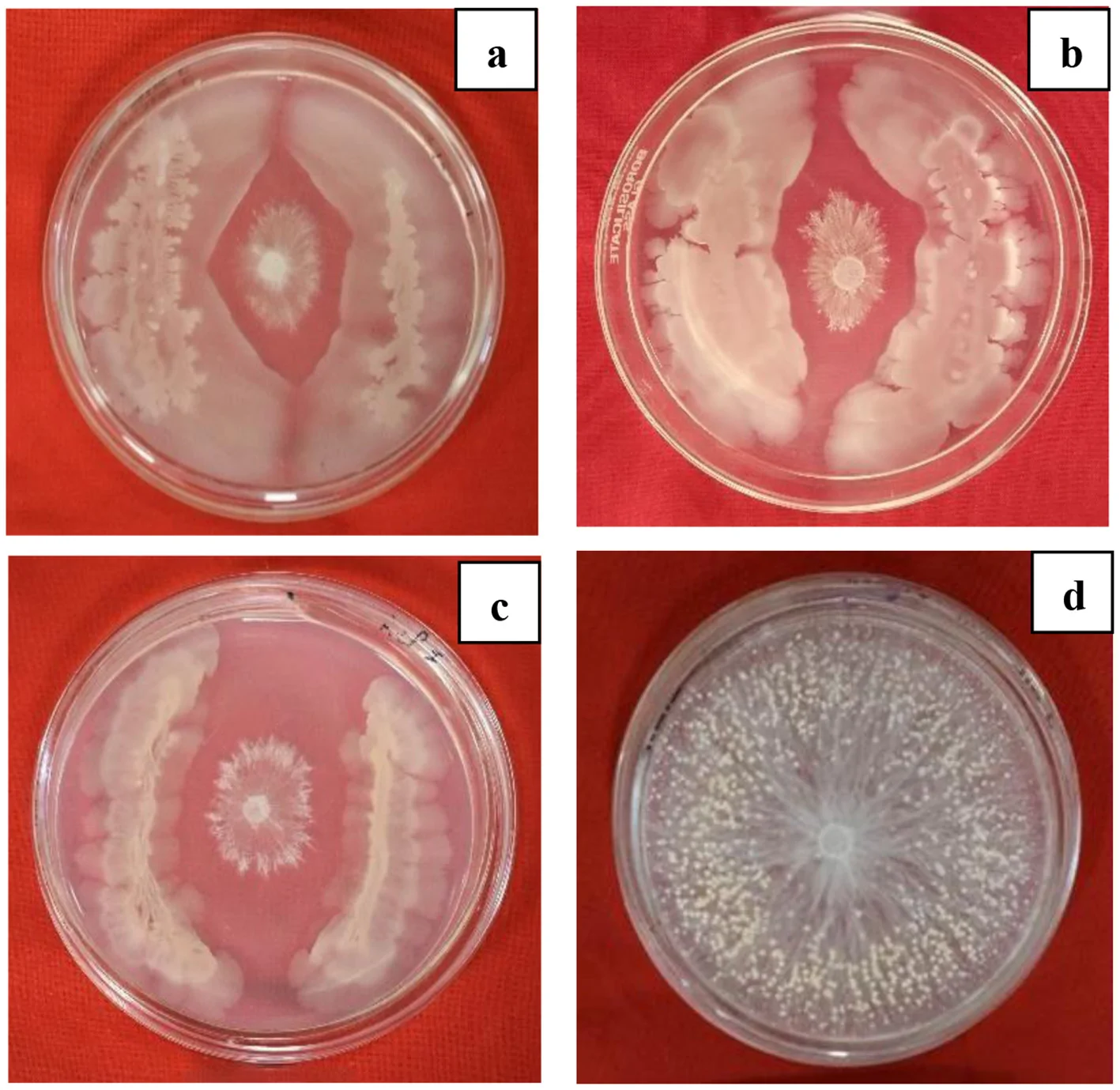
Antagonistic activity of selected endophytic bacterial isolates against *Sclerotium rolfsii* in dual culture assay. **(a)** RLB E-30, **(b)** RLB E-64, **(c)** RLB E-25, and **(d)** pathogen control (*S. rolfsii* grown without bacterial antagonist).

**Table 4:** Antagonistic activity of effective endophytic bacteria against *S. rolfsii* by dual culture assay.

Isolates	Radial growth of *S. rolfsii*							
	24 h (mm)		48 h (mm)		72 h (mm)		96 h (mm)	
	Radial growth (mm)	Inhibition (%)	Radial growth (mm)	Inhibition (%)	Radial growth (mm)	Inhibition (%)	Radial growth (mm)	Inhibition (%)
RLB E-25	11.00	63.33	14.00	70.83	20.33	71.01	20.33	73.71
RLB E-30	08.00	73.33	10.00	79.16	14.00	79.71	15.66	82.60
RLB E-64	10.66	64.46	11.66	75.70	14.66	78.75	19.66	78.15
Control	29.00		48.00		69.00		90.00	
CD (5%)	2.14		2.44		3.65		2.03	
SEm±	0.60		0.69		1.03		0.57	

**Table 5:** Antagonistic activity of effective endophytic bacteria against *S. rolfsii* by inverted plate bioassay.

Isolates	Radial growth of *S. rolfsii*							
	24 h (mm)		48 h (mm)		72 h (mm)		96 h (mm)	
	Radial growth (mm)	Inhibition (%)	Radial growth (mm)	Inhibition (%)	Radial growth (mm)	Inhibition (%)	Radial growth (mm)	Inhibition (%)
RLB E-25	10.00	66.66	15.66	69.88	16.66	68.62	28.66	68.15
RLB E-30	06.00	80.00	06.33	91.34	07.33	89.67	08.33	90.74
RLB E-64	08.60	71.10	11.66	77.57	13.33	81.22	19.33	78.52
Control	30.00		52.00		71.00		90.00	
CD (5%)	1.796		2.352		3.528		5.126	
SEm±	0.509		0.667		1.00		1.45	

### *In vivo* assessment of selected endophytes for growth promotion

Based on the dual culture assay, three best endophytic isolates were selected for further *in-vivo* assessment and further characterization of the bacterial isolates. The three most effective EB that showed potential antagonism against *S. rolfsii* were used for *in vivo* screening in a pot culture study for the observation of germination percentage, pre -emergence mortality (%), post-emergence mortality (%) and total mortality (%). The results of the study indicate a strong correlation between the application of endophytic bacteria (EB) treatments, particularly treatment T5 (Seed priming + Drenching with RLB E-30) and improved plant health parameters across various metrics. The germination rates ranged from 74.06% to 92.58%, with T5 (Seed priming + Drenching RLB E-30) showing the highest germination rate of 92.58%, followed closely by T4 (88.88%). The control (Disease Control) treatment had a relatively low germination rate of 77.77% and the minimum germination rate of 74.06% was observed in T3. These results demonstrate that treatments involving effective endophytic bacteria (EB), particularly T5, enhanced germination rates significantly. Disease incidence, measured through the PDI at 25, 35 and 45 days after sowing (DAS), revealed that T5 consistently had the lowest PDI across all observations (7.87 at 25 DAS, 17.89 at 35 DAS), whereas the highest PDI was recorded in T9 (Soil drenching with RLB E-25) at 33.33 at 35 DAS. The control (Disease Control) treatment had a significantly higher PDI of 33.16. The low PDI in T5 indicates a strong bio-control effect of RLB E-30 against the pathogen, outperforming both the chemical control and other treatments. The analysis of mortality rates further supports the effectiveness of T5. The pre-emergence mortality rate was lowest in T5 (Seed priming + Drenching RLB E-30) and highest in T9 (Soil drenching with RLB E-25) at 25.92%. The control treatment (Disease Control) had a pre-emergence mortality rate of 31.37%. The post-emergence mortality rate followed a similar pattern, with T5 having the lowest post-emergence mortality and T9 the highest at 26.90%, while the control (Disease Control) exhibited 46.42% post-emergence mortality. The total mortality was also minimized in T5 at 19.43%, as compared to the control's (Disease Control) 77.79%, demonstrating a comprehensive reduction in both pre-and post-emergence mortality through the use of endophytic bacteria, particularly RLB E-30 (Table 6; Figures 4, 5).

**Table 6:** Effect of endophytic bacteria on germination percentage, PDI, pre-emergence mortality, post-emergence mortality, and total mortality.

Treatment	Germination (%)	PDI			Pre emergence mortality (%)	Post-emergence mortality (%)	Total mortality (%)
		At 25 days	At 35 days	At 45 days			
T1	77.77	18.04	26.26	33.53	22.13	19.37	41.50
T2	81.47	12.68	24.46	26.08	18.51	14.94	33.45
T3	74.06	16.07	30.26	39.43	25.42	24.60	50.52
T4	88.88	09.33	24.93	26.09	11.11	16.66	27.77
T5	92.58	07.87	17.89	18.23	07.40	12.03	19.43
T6	77.77	15.28	28.90	38.77	20.55	21.38	41.93
T7	79.29	19.37	26.19	35.93	19.69	20.37	40.06
T8	85.17	14.09	23.80	28.86	14.81	17.59	32.04
T9	76.34	20.83	33.33	39.10	25.92	26.90	52.32
T10	80.84	18.49	23.15	35.10	18.45	18.44	36.89
T11	70.36	33.16	47.61	59.32	31.37	46.42	77.79
C.D.	4.96	2.439	3.661	09.44	3.10	05.32	
SE(m)	1.68	0.826	1.240	03.20	1.04	01.80	

T1, Seed priming with EB 1 (RLB E-64); T2, Seed priming + Drenching with EB 1 (RLB E-64); T3, Soil drenching with EB 1 (RLB E-64); T4, Seed priming with EB 2 (RLB E-30); T5, Seed priming + Drenching EB 2 (RLB E-30); T6, Soil drenching with EB 2 (RLB E-30); T7, Seed priming with EB 3 (RLB E-25); T8, Seed priming +Drenching EB 3 (RLB E-25); T9, Soil drenching with EB 3 (RLB E-25); T-10, Seed treatment with Mancozeb @ 3 g/kg seed; T-11, Disease Control (with pathogen and without EB).

**Figure 4:**
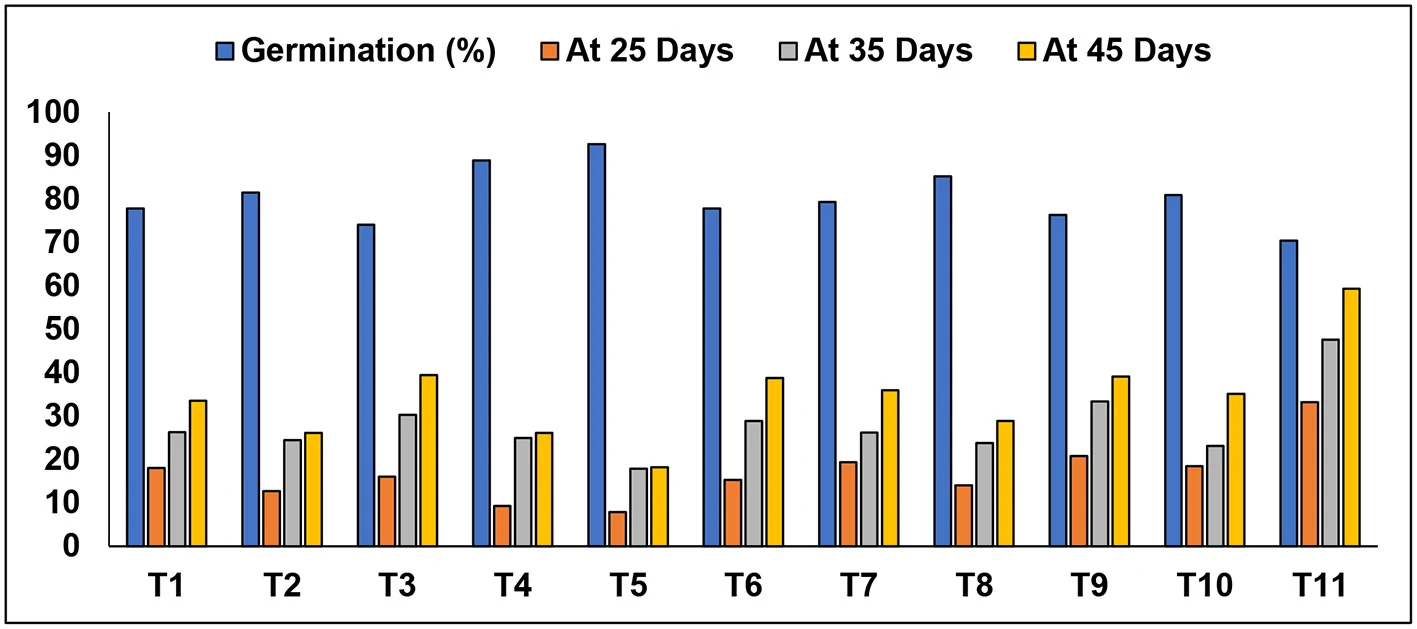
Effect of endophytic bacterial isolates on seed germination (%) and percent disease incidence (PDI) of chickpea challenged with *Sclerotium rolfsii*.

**Figure 5:**
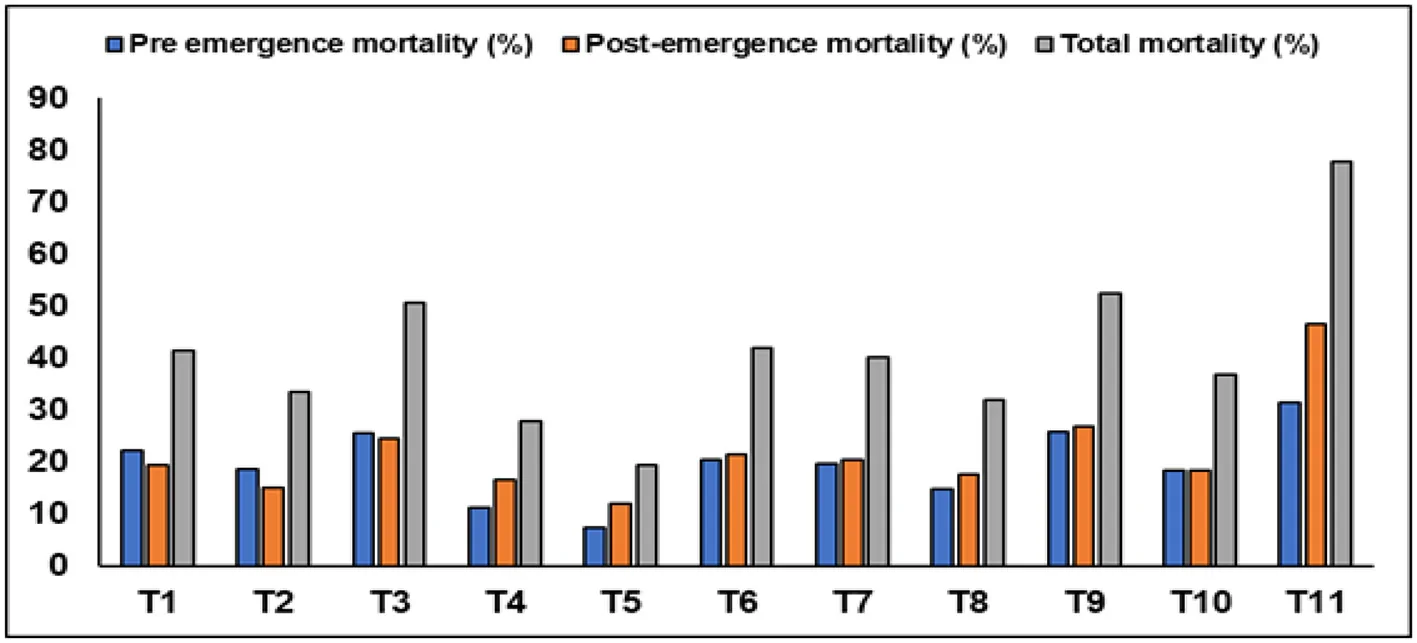
Effect of endophytic bacterial treatments on pre-emergence mortality, post-emergence mortality and total mortality of chickpea caused by *Sclerotium rolfsii* under pot culture conditions.

Data transformation was not performed in CRD experiment because the observed data satisfied the basic assumptions required for analysis of variance (ANOVA). The experimental observations exhibited an approximately normal distribution with homogeneous error variances among treatments and the coefficient of variation was within acceptable limits. So, the original (untransformed) values were used for ANOVA. Therefore, treatment effects were analyzed directly using the appropriate experimental design and mean comparisons were made at the 5% level of significance.

### Biochemical and molecular characterization of the effective bacterial endophytes

#### Biochemical characterization of the effective bacterial endophytes

The biochemical characterization of bacterial isolates as mentioned in Table 7. showed that all strains produced acid from Sucrose, sorbitol, mannitol and glucose. These isolates are identified as *Bacillus velezensis* (*B. velezensis*), *Alcaligenes faecalis* (*A. faecalis*), and *Acinetobacter schindleri* (*A. schindleri*).

**Table 7:** Biochemical characterization of potential bacterial isolates.

Test	RLB E-25	RLB E-30	RLB E-64
Indole	+	+	+
Methyl red	+	+	+
Voges Proskauer's	–	–	–
Citrate utilization	–	–	–
Glucose	+	+	+
Adonitol	–	–	–
Arabinose	–	–	–
Lactose	–	–	–
Sorbitol	+	+	+
Mannitol	+	+	+
Rhamnose	–	–	–
Sucrose	+	+	+

#### Molecular characterization of effective bacterial endophytes

Three bacterial isolates exhibiting maximum potential and enhanced antagonistic activity were successfully isolated from different plant tissues, which were characterized by Sanger sequencing. Genomic DNA was isolated from EB for molecular analysis by CTAB method. Quality and quantity of DNA ranged from 572 to 1,050 ng/μL, was estimated by Nanodrop (Thermo Fischer). 1,500 bp amplified product was Sanger sequenced. Phylogenetic analysis of the 16S rRNA gene sequence revealed high similarity of the isolates with previously reported bacterial species. The sequence analysis of isolate RLB E-25 showed similarity with *Bacillus velezensis* (PX711159.1) with query coverage of 100% and identity of 100% with a maximum score of 1,592. The sequence analysis of isolate RLB E-30 showed similarity with *Alcaligenes faecalis* (PX711247.1) with query coverage of 100% and identity of 100% with maximum score of 2,252 and sequence analysis of isolate RLB E-64 showed similarity with *Acinetobacter schindleri* (PX711283.1) with query coverage of 100% and identity of 100%, with a maximum score of 2,492. The phylogenetic tree analysis revealed that *Bacillus velezensis* RLB E-25 (PX711159.1) and *Bacillus subtilis* were clustered in one group and cluster with *Bacillus velezensis* in the phylogenetic tree (Figure 6). This analysis involved 11 nucleotide sequences. There was a total of 1,485 positions in the final dataset. The phylogenetic tree analysis revealed that *Alcaligenes faecalis* RLB E-30 (PX711247.A) clustered with *Alcaligenes* sp. and *Alcaligenes faecalis* in the phylogenetic tree (Figure 7). This analysis involved 12 nucleotide sequences. There were a total of 1446 positions in the final dataset. The phylogenetic tree analysis revealed that *Acinetobacter schindleri* RLB E-64 (PX711283.1) were clustered with *Acinetobacter schindleri* in the phylogenetic tree (Figure 8).

**Figure 6:**
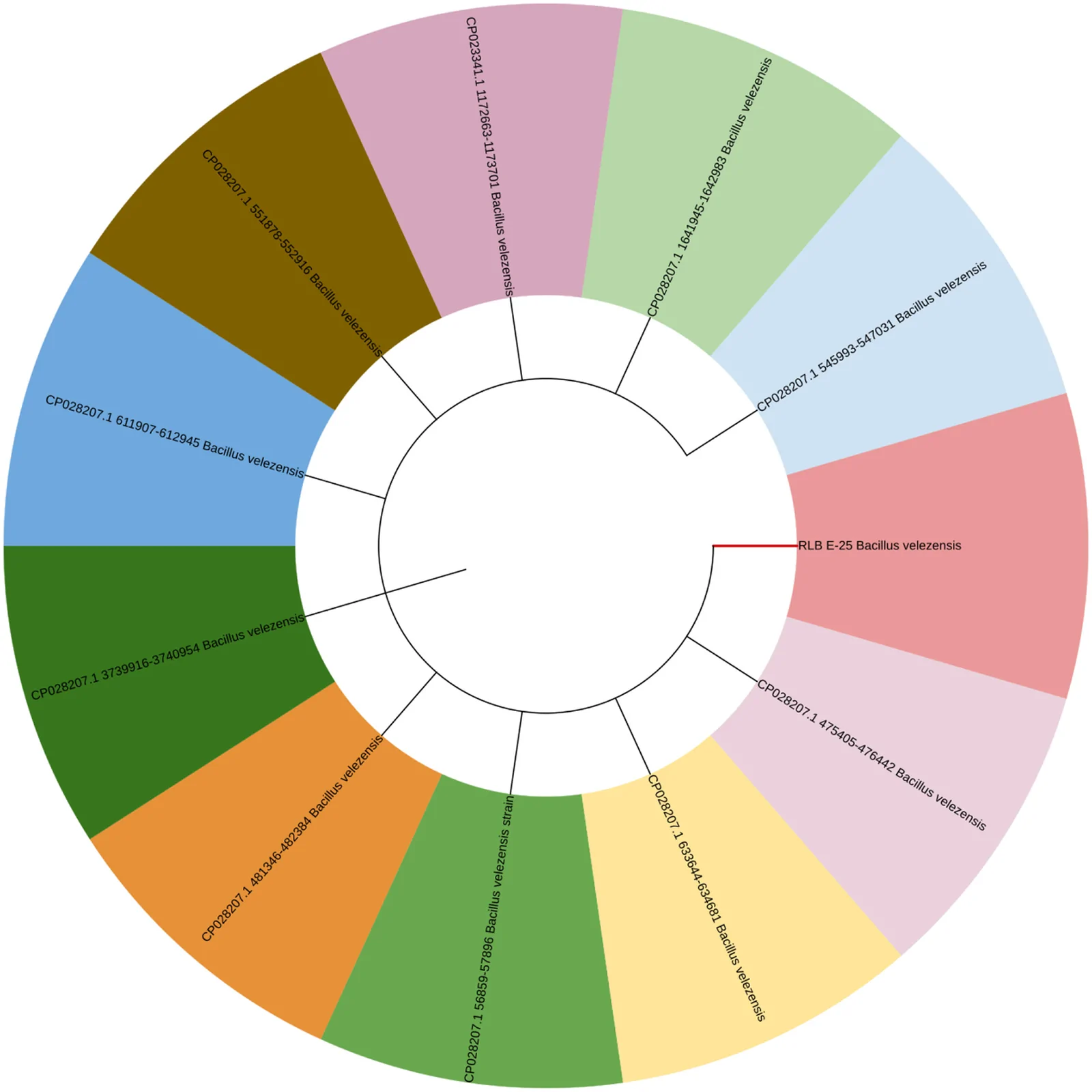
The evolutionary history was inferred using the Neighbor-Joining method. The optimal tree is shown. The percentage of replicate trees in which the associated taxa clustered together in the bootstrap test (1,000 replicates) is shown next to the branches. The evolutionary distances were computed using the Maximum Composite Likelihood method and are in units of the number of base substitutions per site.

**Figure 7:**
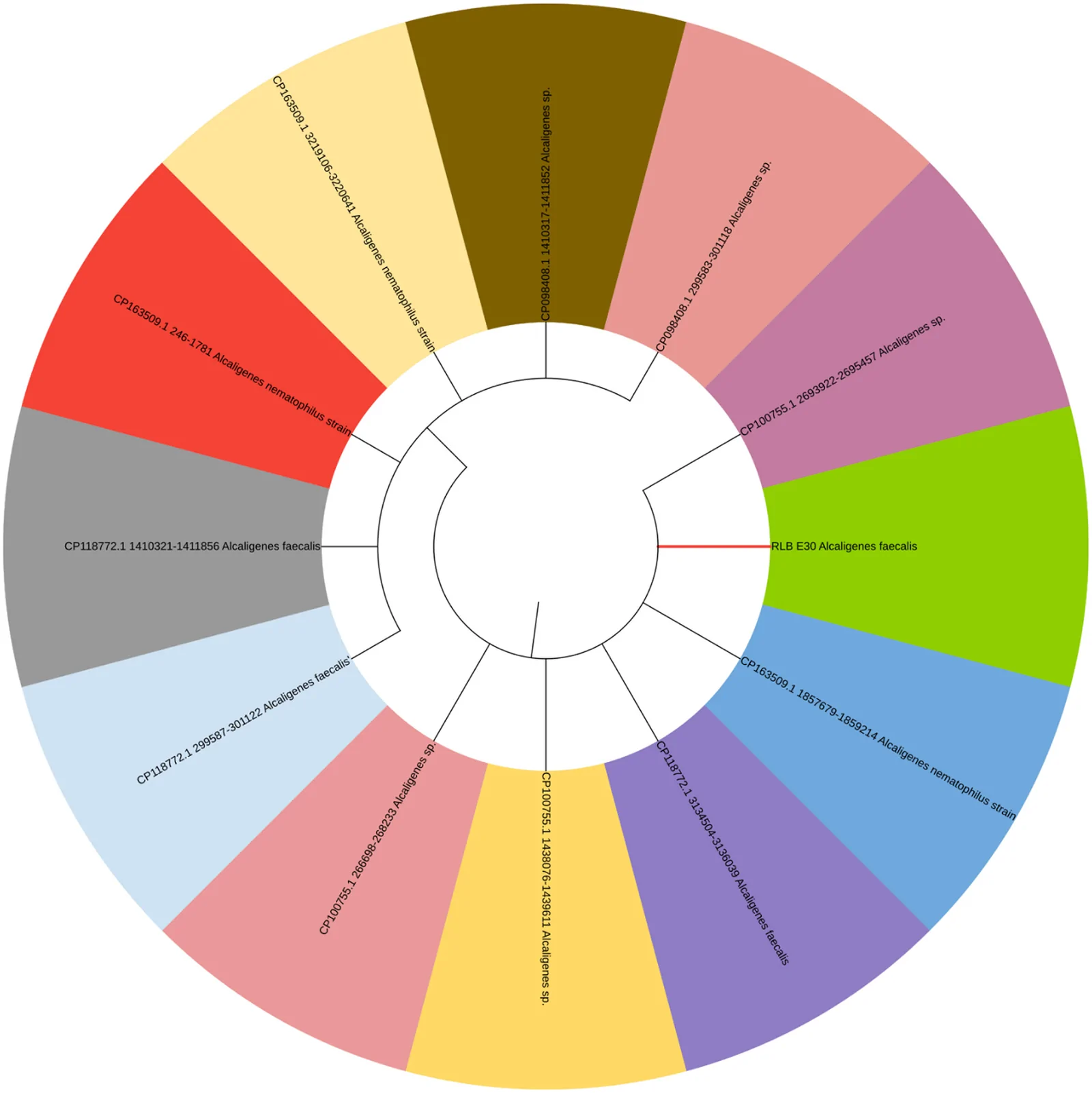
The evolutionary history was inferred using the Neighbor-Joining method. The optimal tree is shown. The percentage of replicate trees in which the associated taxa clustered together in the bootstrap test (1,000 replicates) are shown next to the branches. The evolutionary distances were computed using the Maximum Composite Likelihood method and are in the units of the number of base substitutions per site.

**Figure 8:**
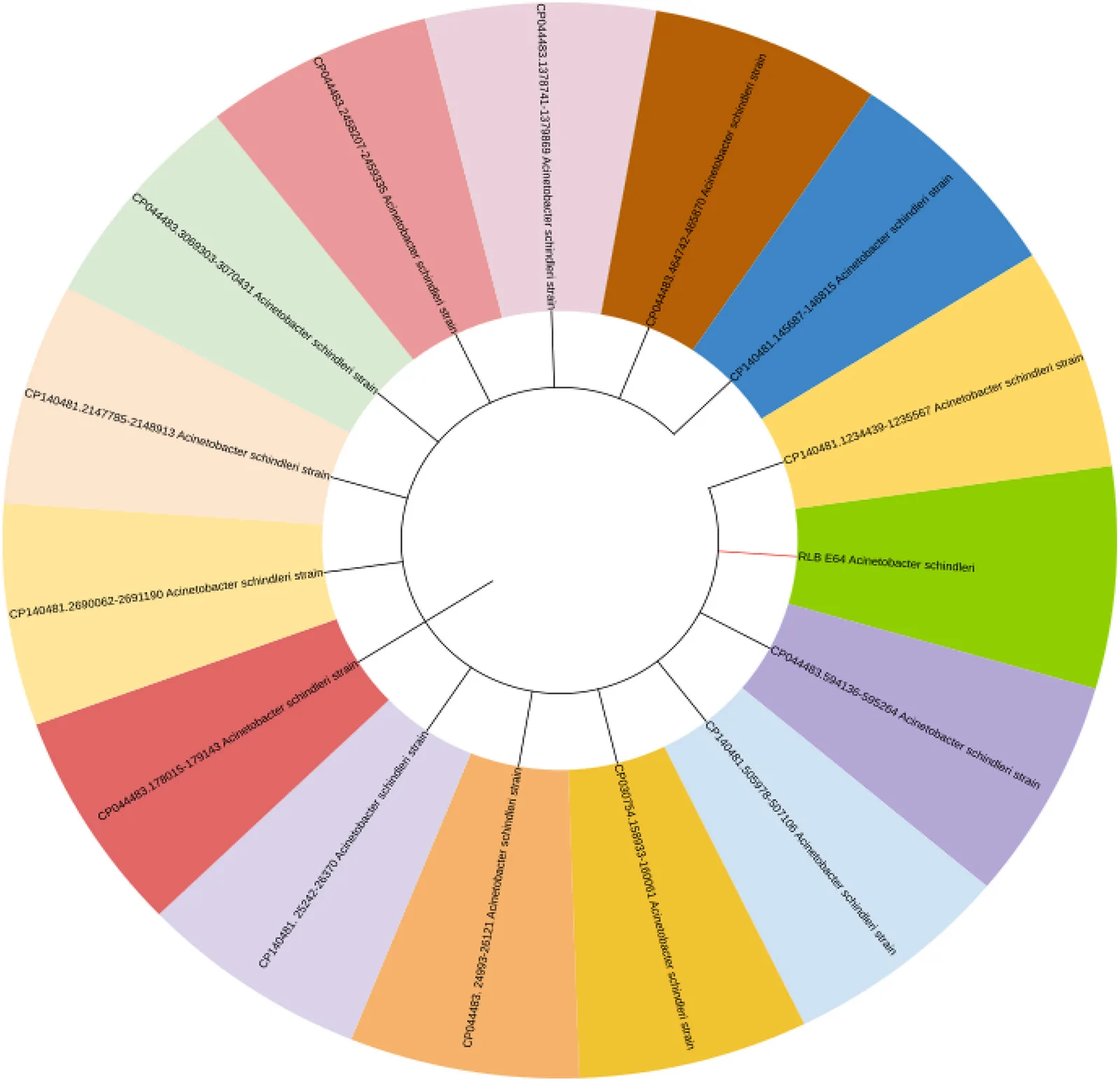
The evolutionary history was inferred using the Neighbor-Joining method. The optimal tree is shown. The percentage of replicate trees in which the associated taxa clustered together in the bootstrap test (1,000 replicates) is shown next to the branches. The evolutionary distances were computed using the Maximum Composite Likelihood method and are in units of the number of base substitutions per site.

### Estimation of defense-related enzymes

In the present investigation, an increased level of defense enzyme activity, such as SOD, POX, APX, CAT and PPO, was recorded. Results of the present experiment gave us a clear idea that all three endophyte bacteria treatments can induce the defense enzymes and can also trigger defense signaling molecules. In the case of non-inoculated treatments, very low activity of defense enzymes was observed (Table 8).

**Table 8:** Level of SOD, POX, APX, CAT, and PPO activity in roots of chickpea.

Treatment	SOD		POX		APX		CAT		PPO	
	Pre inoculation	Post inoculation	Pre inoculation	Post inoculation	Pre inoculation	Post inoculation	Pre-inoculation	Post inoculation	Pre inoculation	Post inoculation
T1	37.25 ± 3.51	77.34 ± 8.21	22.74 ± 2.38	135.62 ± 16.17	7.66 ± 0.78	15.11 ± 2.12	1.02 ± 0.13	6.46 ± 1.02	4.12 ± 0.41	10.13 ± 1.13
T2	43.40 ± 4.85	110.01 ± 11.35	25.29 ± 3.96	137.81 ± 17.61	8.29 ± 0.70	18.74± 2.07	1.02 ± 0.15	7.68 ± 0.62	4.26 ± 0.57	10.08 ± 1.35
T3	35.03± 2.84	99.89± 9.32	30.91 ± 3.94	79.28 ± 8.36	6.93 ± 0.67	21.07 ± 2.76	1.44 ± 0.21	11.96 ± 1.00	4.10 ± 0.42	12.46 ± 1.92
T4	46.20± 6.97	79.65 ± 12.10	23.25 ± 1.53	156.02 ± 17.10	9.48 ± 0.99	20.45 ± 2.04	1.82 ± 0.16	17.06 ± 1.91	4.66 ± 0.54	11.58 ± 1.11
T5	52.90 ± 6.36	191.91 ± 18.86	39.30 ± 6.04	215.11 ± 33.39	19.63 ± 2.54	54.44 ± 5.15	1.95 ± 0.16	23.12 ± 2.92	4.81 ± 0.76	13.07 ± 1.16
T6	42.93 ± 3.97	161.77 ± 24.56	29.79 ± 3.07	102.86 ± 10.36	9.61 ± 0.98	22.56 ± 2.70	1.89 ± 0.29	18.63 ± 1.67	4.52 ± 0.57	10.21 ± 0.97
T7	41.51 ± 3.47	164.44 ± 20.82	25.29 ± 2.83	51.65 ± 5.98	12.49 ± 1.62	24.19 ± 3.37	1.91 ± 0.19	12.87 ± 1.12	4.60 ± 0.55	11.74 ± 1.29
T8	46.20 ± 6.34	136.83 ± 16.16	21.88 ± 2.5	87.67 ± 8.15	11.88± 1.82	28.59 ± 4.39	1.61 ± 0.18	8.55 ± 0.68	4.73 ± 0.61	12.46 ± 1.42
T9	33.86 ± 3.85	126.52 ± 16.08	18.83 ± 2.29	37.16 ± 3.97	8.12 ± 0.80	12.04 ± 1.01	1.04 ± 0.13	9.50 ± 1.31	3.78 ± 0.52	7.03 ± 0.42
T10	43.34 ± 1.13	111.43 ± 1.10	20.63 ± 3.29	98.63 ± 9.49	8.55 ± 0.36	26.00 ± 3.93	1.61 ± 0.19	2.42 ± 0.36	4.23 ± 0.51	5.78 ± 0.47
T11	13.03± 3.01	7.40 ± 1.05	13.25 ± 1.93	8.34 ± 1.25	6.67 ± 3.97	7.61 ± 1.02	0.89 ± 0.08	0.94 ± 0.10	2.38 ± 0.51	3.49 ± 0.72

The SOD activity was measured before and after pathogen inoculation conditions. In Pre-inoculation, SOD activities increased from 33.86 ± 3.85 to 52.90 ± 6.36 U mg/protein, with the highest activity in T5 and the lowest in T9. The control recorded significantly lower activity at 13.03 ± 3.01 U mg/protein. While in post-inoculation conditions, SOD activity was consistently higher in post-inoculation compared to the activity of pre-inoculation, ranging from 126.52 ± 16.08 to 190.91 ± 18.86 U mg/protein. The maximum activity was again observed in T5, while T9 showed the lowest. The control (T11) exhibited minimal change, with 13.25 ± 1.93 U mg/protein. The activity of defense enzymes increased from pre-inoculation to post-inoculation, indicating a significant induction of defense-related biochemical response following pathogen challenge (Figures 9, 10).

**Figure 9:**
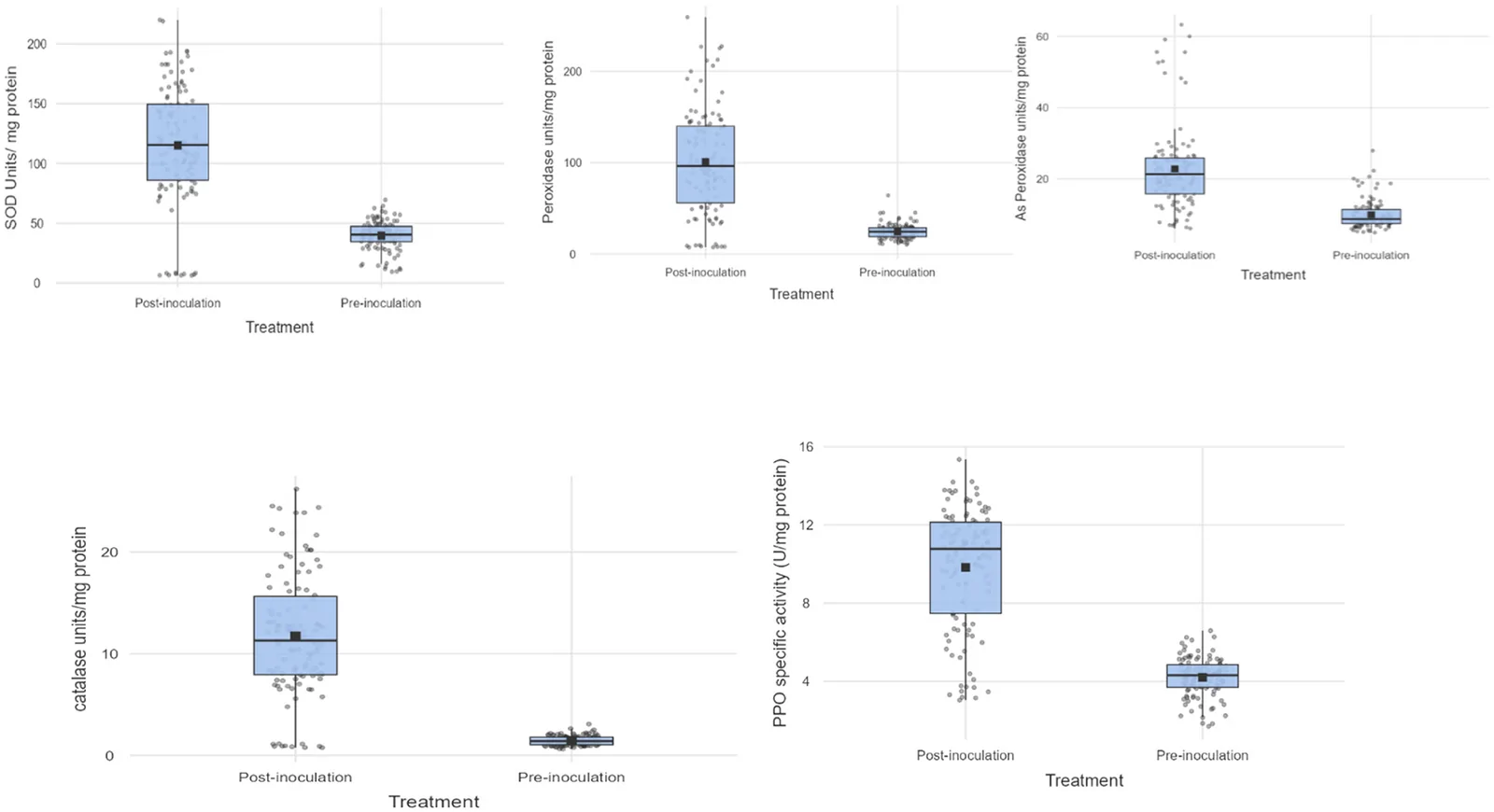
Box plots illustrating the distribution of SOD, POX, APX, CAT, and PPO activities across different treatments in chickpea challenged with *Sclerotium rolfsii*.

**Figure 10:**
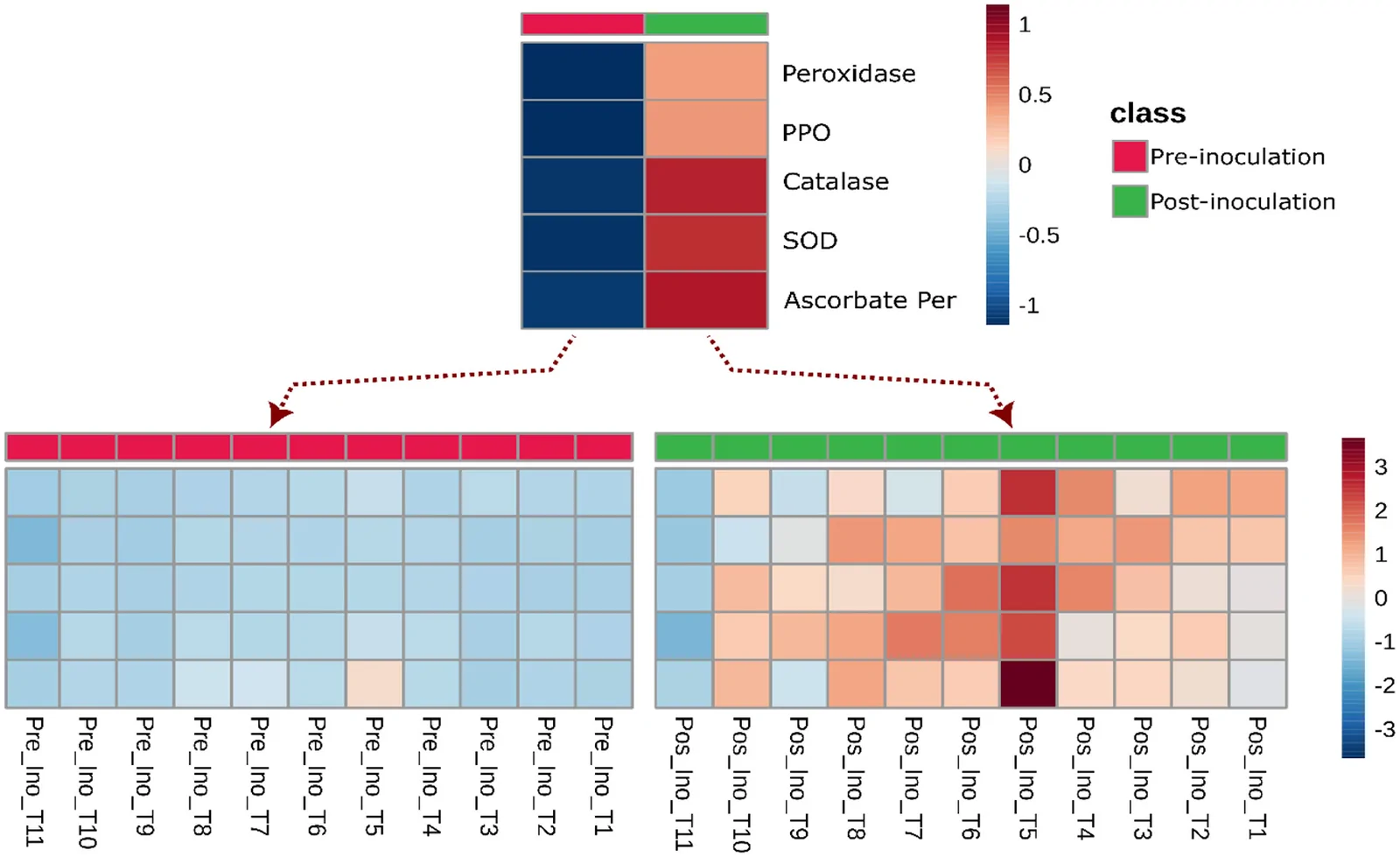
Heat map depicting differential expression of defense-related enzymes in chickpea following inoculation with endophytic bacteria under pathogen-challenged and non-challenged conditions.

### Peroxidase (POX)

POX activity was measured across treatments pre- and post-inoculation. POX activity in pre-inoculation ranged from 18.83 ± 2.29 to 39.30 ± 6.04 U mg/protein. The highest activity was observed in T5 (Seed priming + Drenching with RLB E-30), while the lowest was in T9 (Soil drenching with RLB E-25). The control (T11) recorded 13.25 ± 1.93 U mg/protein. Other treatments showed moderate POX activity levels. Post-inoculation POX activity increased significantly, ranging from 37.16 ± 3.97 to 215.11 ± 33.39 U mg/protein. T5 again showed the highest activity, while T9 had the lowest. The control (T11) exhibited minimal activity at 8.34 ± 1.25 U mg/protein. Other treatments demonstrated varying increases, with notable activity in T1, T2 and T4. These results highlight the enhanced POX activity in response to inoculation, particularly in treatment T5, indicating its role in systemic resistance (Figures 9, 10).

### Ascorbate peroxidase (APX)

APX activity was measured across all treatments, both pre- and post-inoculation. During pre-inoculation, APX activity values ranged from 3.93 ± 0.67 to 19.63 ± 2.54 U mg/protein, with the control (T11) showing an activity level of 6.67 ± 3.97 U mg/protein. The highest pre-inoculation APX activity was observed in treatment T5 (Seed priming + Drenching Endophyte RLB E-30), which recorded 19.63 ± 2.54 U mg/protein, while the lowest value, 3.93 ± 0.67 U mg/protein, was recorded in treatment T3 (Soil Drenching RLB E-25). Post-inoculation, APX activity increased significantly, with values ranging from 12.04 ± 1.01 to 55.44 ± 5.15 U mg/protein, while the control (T11) had a lower activity level of 7.61 ± 1.02 U mg/protein. The highest post-inoculation APX activity was recorded in treatment T5 (Seed priming + Drenching RLB E-30), with 55.44 ± 5.15 U mg/protein and the lowest value was observed in T9 (Soil drenching RLB E-25), with 12.04 ± 1.01 U mg/protein (Figures 9, 10).

### Catalase activity (CAT)

CAT activity was measured across all treatments, both pre- and post-inoculation. During pre-inoculation, CAT activity values ranged from 1.02 ± 0.13 to 1.95 ± 0.16 U mg/protein, with the control (T11) showing an activity level of 0.89 ± 0.08 U mg/protein (Table 5). The highest pre-inoculation CAT activity was observed in treatment T5 (Seed Priming + Drenching with RLB E-30), which recorded 1.95 ± 0.16 U mg/protein, while the lowest value, 1.02 ± 0.13 U mg/protein, was recorded in treatment T1 (Seed Priming with RLB E-64). Post-inoculation, CAT activity significantly increased, with values ranging from 2.42 ± 0.36 to 23.12 ± 2.92 U mg/protein, while the control (T11) showed a lower activity level of 0.94 ± 0.10 U mg/protein. The highest post-inoculation CAT activity was recorded in treatment T5 (Seed priming + Drenching with RLB E28), with 23.12 ± 2.92 U mg/protein and the lowest value was observed in T10 (Seed treatment with Mancozeb), with 2.42 ± 0.36 U mg/protein (Figures 9, 10).

### Polyphenol oxidase (PPO)

PPO activity was analyzed for all treatments, both pre- and post-inoculation. During pre-inoculation, PPO activity values ranged from 3.78 ± 0.52 to 4.81 ± 0.76 U mg/protein, with the control (T11) showing an activity level of 2.38 ± 0.51 U mg/protein (Table 5 and Figure 9). The highest pre-inoculation PPO activity was observed in treatment T5 (Seed priming + drenching RLB E-30), which recorded 4.81 ± 0.76 U mg/protein, while the lowest value, 3.78 ± 0.52 U mg/protein, was recorded in treatment T9 (Soil Drenching with RLB E-25). Post-inoculation, PPO activity increased significantly, with values ranging from 5.78 ± 0.47 to 13.07 ± 1.16 U mg/protein, while the control (T11) showed an activity level of 3.49 ± 0.72 U mg/protein. The highest post-inoculation PPO activity was recorded in treatment T5 (Seed priming + Drenching with RLB E-30) with 13.07 ± 1.16 U mg/protein and the lowest value was observed in T10 (Seed treatment with Mancozeb), with 5.78 ± 0.47 U mg/protein (Figures 9, 10).

Since the observed data was in the range of fundamental requirements for analysis of variance (ANOVA), data transformation was not done in the CRD experiment conducted for defense enzyme analysis. The coefficient of variation was within allowable range and the experimental observations showed a roughly normal distribution with uniform error variances across treatments. Thus, the ANOVA was performed using the original (untransformed) results. As a result, mean comparisons were established at the five percent level of significance and treatment effects were directly recorded utilizing the proper experimental design.

## Discussion

The wide and extensive use of chemical fungicides for managing soil-borne diseases has made several researchers and people about environmental contamination and also pathogen resistance along with risks to human and animal health (Shekhar et al., 2025). Therefore, biological control using endophytic bacteria has become a more sustainable alternative as these microorganisms colonize the internal tissues of plant and then suppress pathogens via several complementary mechanisms as they support plant growth (Afzal et al., 2019). Unlike chemical fungicides, the endophytes build more persistent connections with the host, as protection keeps lasting through diverse mechanisms like nutrient competition, antimicrobial metabolite production, induction of systemic resistance (ISR) and also improvement in nutrient acquisition (Oukala et al., 2021).

In the current study, out of 73 bacterial endophytes screened against *S. rolfsii*, three potential endophytic bacteria *viz., Alcaligenes faecalis* (RLB E-30), *Bacillus velezensis* (RLB E-25), and *Acinetobacter schindleri* (RLB E-64) showed maximum antagonism which was contributed by the simultaneous mechanism of antibiotics, lytic enzymes, volatile organic compounds (VOCs) and biofilm formation, by inhibiting pathogen growth and colonization of root by the pathogen. Similar reports have been made in chickpea, where *S. rolfsii* and *Fusarium oxysporum* f. sp. *ciceri* were inhibited by endophytic *Bacillus* spp. and *Pseudomonas* spp. by releasing anti-fungal metabolites (Mageshwaran et al., 2022; Zou et al., 2022; Likhita et al., 2026). Singh et al. (2022) and Abdullaeva et al. (2024) further demonstrated that these endophytic bacteria have ability to colonize inside plant tissues by establishing stable populations within chickpea roots, thereby reducing the incidence of disease incidence by providing prolonged protection.

The biocontrol efficacy of the selected endophytic bacterial isolates against *S. rolfsii*, was further evaluated under pot culture conditions. Among the treatments, T5 (seed priming + soil drenching with RLB E-30) recorded the highest germination percentage (92.58%) and the lowest pre- and post-emergence mortality of indicating the superior antagonistic ability of this isolate against the pathogen. The lower total mortality (19.43%) in T5 was minimum when compared with the untreated control (77.77% germination and 77.79% total mortality), which clearly demonstrate the effectiveness of endophytic bacteria in enhancing plant establishment and suppressing disease incidence under controlled conditions. Similar observations in seed germination, seedling vigor and plant growth following endophytic bacterial inoculation under pathogen pressure have been reported earlier by Sahu et al. (2020) in tomato challenge inoculated with *S. rolfsii*. The consistent lower per cent disease incidence (PDI) recorded in T5 at all observation intervals (7.87 at 25 DAS and 17.89 at 35 DAS) further confirm the strong suppressive effect of the endophytic bacterium against *S. rolfsii*. Comparable reductions in disease incidence have been reported with bacterial antagonists such as *Bacillus velezensis* and *Pseudomonas fluorescens* against *S. rolfsii* in crops like groundnut and tomato (Li et al., 2023).

The superior performance of isolate RLB E-30 (*Alcaligenes faecalis*) can be attributed to the involvement of multiple biocontrol mechanisms, including the production of antifungal metabolites, cell wall–degrading enzymes and volatile organic compounds that collectively inhibit pathogen growth and enhance host resistance. Recent studies have emphasized the role of *Bacillus* spp. derived lipopeptides such as fengycins, iturins, and surfactins in suppressing soil-borne pathogens through membrane disruption and inhibition of mycelial growth (Khan et al., 2020). Additionally, the induction of defense-related enzymes, including peroxidase, phenylalanine ammonia-lyase and polyphenol oxidase, along with increased accumulation of phenolic compounds, contributes significantly to enhanced resistance in endophyte-treated plants, as reported by Singh et al. (2022) in chickpea infected with *F. oxysporum* f. sp. *ciceri*.

The enhanced plant growth parameters recorded in T5 treatment further supports the plant growth-promoting role of EB. Treated plants exhibited healthier and more robust root architecture, which are crucial for efficient nutrient uptake and increased mechanical resistance to pathogen invasion. Similar enhancements in root growth parameters, including root length, diameter and volume, have been reported in wheat plants inoculated with *Bacillus subtilis* and *Arthrobacter* spp. (Aijaz et al., 2024), as well as in chickpea inoculated with endophytic bacteria under pathogen stress (Abdullaeva et al., 2024).

The biochemical characterization of the three most effective endophytic bacterial isolates revealed their metabolic versatility as evidenced by positive reactions for indole production, methyl red test and utilization of glucose, sorbitol, mannitol and sucrose. Variations in sugar fermentation patterns among isolates were consistent with descriptions provided in Bergey's Manual of Systematic Bacteriology (Vos et al., 2011). Similar metabolic diversity was described among chickpea-associated *Bacillus* and *Alcaligenes* isolates with strong biocontrol potential (Ray et al., 2023). Such biochemical variability may arise due to the gain or loss of transferable genes, particularly those encoded on plasmids that are responsible for carbohydrate metabolism, leading to metabolic inconsistencies among closely related strains (Ahrné et al., 1989).

Molecular identification confirmed that the three endophytic isolates belonged to *Bacillus velezensis, Alcaligenes faecalis*, and *Acinetobacter schindleri*. These findings are in agreement with earlier reports indicating that members of the genus *Bacillus* constitute a major proportion of effective endophytic biocontrol agents across diverse crops (Sun et al., 2013; Chen et al., 2014; Zhao et al., 2015; Kumar et al., 2016), as well as *Alcaligenes faecalis* (Ray et al., 2023). Similar antagonistic activity against *S. rolfsii* has been reported for *Bacillus pumilus* (Archana et al., 2020) and *Alcaligenes faecalis* (Ray et al., 2016), further supporting the relevance of the isolates identified in this study.

The enhanced activities of the key defense-related enzymes *viz*. SOD, POX, APX, CAT and PPO in endophyte-treated chickpea plants, particularly under T5 treatment, highlighted the role of bacterial endophytes in activating host defense mechanisms. Enzyme activities increased consistently from pre- to post-pathogen inoculation stages, indicating a rapid and effective defense response upon pathogen challenge. The significantly higher enzyme activities observed in T5, compared with the control and soil drenching treatment (T9), suggest that RLB E-30 effectively strengthened the plant's antioxidant defense system along with basal defenses by detoxifying reactive oxygen species and reinforcing induced systemic resistance.

Similar enhancement of antioxidant and defense-related enzymes in chickpea following endophytic bacterial treatment has been reported (Kandoliya and Vakharia, 2015; Egamberdieva et al., 2017). *Bacillus subtilis* has been shown to increase SOD and CAT activities, which play a critical role in mitigating oxidative stress by converting superoxide radicals into less harmful molecules (Egamberdieva et al., 2017). POX and PPO are integral components of the plant defense system and their upregulation following endophytic bacterial inoculation contributes to enhanced resistance against pathogen-induced oxidative stress (Mohammed et al., 2026). Furthermore, endophytic bacteria are known to modulate the expression of genes involved in antioxidant pathways, leading to increased accumulation of antioxidants and improved stress tolerance (Nie et al., 2024).

Consistent with these findings, Ray et al. (2023) reported that *Alcaligenes faecalis* BHU-12 releases bacterial phenolics that enhance host defense responses against pathogens. Similarly, Ray et al. (2016) demonstrated that endophytic *Alcaligenes* spp. activate a wide range of defense responses in okra against *S. rolfsii*, including induction of phyto-peroxidases and activation of the phenylpropanoid pathway, resulting in increased phenolic content. Enhanced resistance has also been associated with systemic induction of phenolics and upregulation of antioxidant enzymes such as peroxidase and superoxide dismutase, leading to increased lignin deposition and strengthened cell walls (Ray et al., 2020).

When compared to *Bacillus velezensis* and *Acinetobacter schindleri, Alcaligenes faecalis* performed better since it has several plant growth-promoting and biocontrol mechanisms. *A. faecalis* is known to produce phytohormones, siderophores, hydrolytic enzymes, and volatile organic chemicals that promote root development, nutrient uptake and induced systemic resistance in addition to potent antagonistic activity against the pathogen. On the other hand, while *B. velezensis* and *A. schindleri* are also known to have biocontrol potential, their efficacy may have been limited in comparison to *A. faecalis* due to variations in rhizosphere colonization efficiency, metabolite production, environmental adaptability and compatibility with the host plant. The fact that the trials were carried out in controlled pot and greenhouse conditions where environmental factors like temperature, humidity, soil moisture and pathogen pressure were comparatively steady, is one of the study's limitations. The complexity and unpredictability of commercial field environments, where soil heterogeneity, climate variations, microbial competition and crop management techniques can affect the establishment and effectiveness of introduced endophytes, are not adequately captured by these conditions. Therefore, to confirm the consistency, durability, and practical relevance of *A. faecalis* as a biocontrol and plant growth-promoting agent under natural production systems, long-term, multi-location field studies across several agroclimatic areas are needed.

### Conclusion

The current study demonstrate that the bacterial endophytes can act as an eco-friendly and good alternative to chemical fungicides for controlling the collar rot disease in chickpea by *S. rolfsii*. Out of the 73 endophytic bacterial isolates, *Alcaligenes faecalis* RLB E-30 strain was found to be the most promising biocontrol agent in terms of its antagonistic potential followed by *Bacillus velezensis* and *Acinetobacter schindleri* based on *in vitro* antagonism, pot culture screening biochemical and molecular characterization. Seed priming along with soil drenching of *Alcaligenes faecalis* greatly improved seed germination by reducing pre-and post-emergence mortalities with lower disease incidence and increased overall plant growth when compared with untreated controls. Such superior performance of this isolate is primarily due to its multifaceted mode of action, including direct antagonism of the pathogen and indirect stimulation of the host defense responses.

Molecular identification confirmed the most promising isolates as *Alcaligenes faecalis, Bacillus velezensis*, and *Acinetobacter schindleri*, which were well-documented for their biocontrol and plant growth promoting attributes aligning with the already reported works. The pronounced induction of antioxidant and defense-related enzymes *viz.*, SOD, POX, APX, CAT and PPO in endophyte-treated plants, particularly under *Alcaligenes faecalis* RLB E-30 strain, highlights the role of these bacteria in strengthening the plant's oxidative defense system and inducing systemic resistance. Improved root development and vigor might also contribute to enhanced nutrient acquisition and minimizes the invasion by the pathogen. Altogether, the conclusion confirms that the possible utilization of endophytic bacteria, especially *Alcaligenes faecalis* (RLB-E 30), as biological control agent against *S. rolfsii* in chickpea.

## Data Availability

The datasets generated and analyzed during the current study are publicly available in the National Center for Biotechnology Information (NCBI) repositories. The 16S rRNA gene sequences generated in this study have been deposited in the NCBI GenBank database under the following accession numbers: PX710924.1 (RLB E-1), PX711159.1 (RLB E-25), PX711247.1 (RLB E-28), PX710790.1 (RLB E-33), and PX711283.1 (RLB E-64). The sequences are publicly available in the NCBI GenBank database. The datasets can be accessed through the NCBI repositories using the corresponding accession numbers.
